# Longitudinal effects of meditation on brain resting-state functional connectivity

**DOI:** 10.1038/s41598-021-90729-y

**Published:** 2021-05-31

**Authors:** Zongpai Zhang, Wen-Ming Luh, Wenna Duan, Grace D. Zhou, George Weinschenk, Adam K. Anderson, Weiying Dai

**Affiliations:** 1 Department of Computer Science, State University of New York at Binghamton, 4400 Vestal Pkwy E, Binghamton, NY 13902 USA; 2 National Institute on Aging, National Institute of Health, Baltimore, MD 21225 USA; 3 Department of Human Development, Cornell University, Ithaca, NY 14853 USA

**Keywords:** Neuroscience, Medical research

## Abstract

Changes in brain resting-state functional connectivity (rsFC) were investigated using a longitudinal design by following a 2-month focused attention meditation (FAM) practice and analyzing their association with FAM practice time. Ten novice meditators were recruited from a university meditation course. Participants were scanned with a resting-state fMRI sequence with multi-echo EPI acquisition at baseline and at the 2-month follow-up. Total FAM practice time was calculated from the daily log of the participants. We observed significantly increased rsFC between the posterior cingulate cortex (PCC) and dorsal attention network (DAN), the right middle temporal (RMT) region and default mode network (DMN), the left and right superior parietal lobules (LSPL/RSPL) and DMN, and the LSPL/RSPL and DAN. Furthermore, the rsFC between the LSPL and medial prefrontal cortex was significantly associated with the FAM practice time. These results demonstrate increased connectivity within the DAN, between the DMN and DAN, and between the DMN and visual cortex. These findings demonstrate that FAM can enhance the brain connection among and within brain networks, especially DMN and DAN, indicating potential effect of FAM on fast switching between mind wandering and focused attention and maintaining attention once in the attentive state.

## Introduction

The Practice of meditation has been associated with altered neural activity and reorganization (see a review^1^). Most examinations are task related, especially those that include meditation or engage trained skills, and are susceptible to expectancy and knowledge-based biases that can change neural signals. Brain activations observed during meditation provide evidence for increases of neural activity while practicing meditation; however, the findings may have been influenced by the individual variance of performing the required meditation during the fMRI scanning. Only when the state of neural activity has increased through repeated meditation practice, when meditation is not being practiced such as during rest, and has become a trait of the brain, will it provide a neural basis for enhanced attentional control and emotional regulation that can translate to daily life. To address how meditation training alters trait-level neural reorganization, we conducted a longitudinal examination of how 8 weeks of Focused Attention Meditation (FAM) training alters task-independent resting-state functional connectivity using multi-echo functional magnetic resonance imaging.

Meditation comes in many forms, such as focused attention or open monitoring or non-dual awareness. Here we examined FAM training, which involves reorienting attention onto an internal or external object and inhibiting distraction of mind wandering or random thoughts. Mind wandering and reorienting attention were associated with neural activity in the default mode network (DMN)^2–6^ and dorsal attention network (DAN)/ventral attention network (VAN)^7–10^, respectively.

FAM has been reported with generally increased task-related neural activation in brain regions that have been reported involved in brain attention processing^11–14^, although they are not completely consistent. Experienced meditators exhibited increased activation during meditation in the anterior cingulate and medial prefrontal cortex (prefrontal regions in DMN) compared to controls^11^. Long-term meditators showed significantly more activation in the majority of attention regions of interest (a priori DAN regions from a meta-analysis involving attention-shift paradigms) than novice meditators during meditation^12^. This study also reported that long-term meditators had more activation in the left superior frontal gyrus and middle frontal gyrus (prefrontal regions in DMN) when compared to incentive novice meditators (to remove the potential motivational difference of the long-term meditators). Similar findings extend to individuals randomized to an 8-week course in mindfulness meditation training, which was associated with decreased cortical midline recruitment associated with internal self-referential process (DMN areas)^13^ and increased activity in brain regions implicated in external visual attention, including inferior and superior parietal lobes (DAN areas) and middle occipital gyrus (Visual Cortex)^14^.

Structural magnetic resonance imaging (sMRI) has associated FAM with trait changes of the brain structure. A number of sMRI studies found increased gray matter density^15–17^ and cortical thickness^18–21^ in brain regions involving self-referential and attention processing, such as superior parietal gyri (posterior regions in DAN), precuneus (a posterior region in DMN), anterior cingulate, superior and middle frontal gyri, and orbitofrontal (prefrontal regions in DMN) regions in meditators. Longitudinal sMRI studies demonstrated the increases in gray matter density after an 8-week meditation training, which provide more direct evidence of the effects of meditation on brain structural changes in posterior cingulate cortex and temporoparietal junction (posterior regions in DMN)^20,22,23^. One of the longitudinal sMRI studies found that reductions in perceived stress were correlated with changes in gray matter density, demonstrating that neuroplastic changes induced by meditation are associated with improvements in mental states.

Brain resting-state functional connectivity (rsFC) can provide the trait measure for coherent neural activity between different regions of the brain during the resting state. Therefore, rsFC can provide a less biased account of neural network changes with meditation practice compared to task-based fMRI. Meditation studies in functional connectivity have conducted cross-sectional studies to compare the rsFC between meditators and controls^24–26^ and the meditation-state functional connectivity (msFC) between meditators and controls^26,27^. Other studies compared the same subjects at different conditions: rsFC and msFC in experienced meditators^24,28^. These cross-sectional resting-state and state-related meditation studies generally involved the changes of functional connectivity in two brain networks: the DMN and DAN, although the directions of changes may not agree well. Longitudinal studies have been performed but mainly focused on the potential benefits of meditation on specific medical groups, such as cognition of the elderly^29^, anxiety of the stressed community^30^, and symptoms of posttraumatic stress disorder (PTSD) subjects^31^. Therefore, the causal effect of meditation on the trait rsFC measure of novice meditators has not been systematically studied.

Consistent with the FAM training process and meditation results from morphological^18–23^ and resting-state rsFC^24–26^ changes, our first hypothesis was that FAM would result in stronger connection within the DAN and/or between the DAN and the visual cortex, for maintaining attentive status over a long period of time. In addition, there would be a stronger connection between DMN and DAN/visual cortex for switching between mind wandering and focused attention. We further hypothesized that rsFC would vary among participants according to the practice time of meditation. While amount of adherence to a meditation practice may reflect an expectancy bias during a task or meditative state during scanning, changes in task-free resting connectivity are less susceptible to these biases and would better reflect baseline changes in brain dynamics associated with and potentially caused by greater practice. We tested the hypotheses by using a longitudinal study design and multi-echo (ME) BOLD fMRI to assess the changes in brain rsFC after practicing FAM over a 2-month period. ME BOLD fMRI, instead of frequently used single-echo BOLD fMRI, has been adopted in the study because of its effectiveness in mitigating imaging artifacts due to subject motion and various sources on physiological noises and thereby increasing SNR^32,33^. This noise is particularly relevant to the meditation studies as there are often peripheral physiological changes in respiration and heart rate and their variability with meditation practice^34^, which can differentially influence detection of BOLD signal and estimates of functional connectivity at baseline and the 2-month follow-up. We expect that the ME fMRI technique will largely increase the reliability of our longitudinal mediation results.

## Results

### Basic characteristics of the participants

The demographic characteristics of all the participants, including age, gender, handedness and meditation practice time are shown in Table 1. All the participants were either 19 or 20 years old. The practice duration and practice time for the ten participants between the baseline and follow up scans is 66.50 ± 4.14 days and 574.00 ± 465.55 min. Two subjects had practice time more than 1000 min.

**Table 1:** Basic characteristics and meditation practice time of subject.

Subject ID	Age(years)	Gender	Handedness	Practice time (min)
101	19	Female	Right	135
102	20	Male	Left	1080
103	19	Male	Right	1620
104	19	Male	Right	400
105	19	Male	Right	360
106	19	Female	Right	720
107	19	Male	Right	160
108	20	Male	Left	495
109^a^	19	Female	Right	N/A
110	19	Female	Left	210
111	19	Female	Right	560

^a^Stands for the subject who was not scanned for follow-up.

### Differences in rsFC between baseline and follow-up

**Figure 1:**
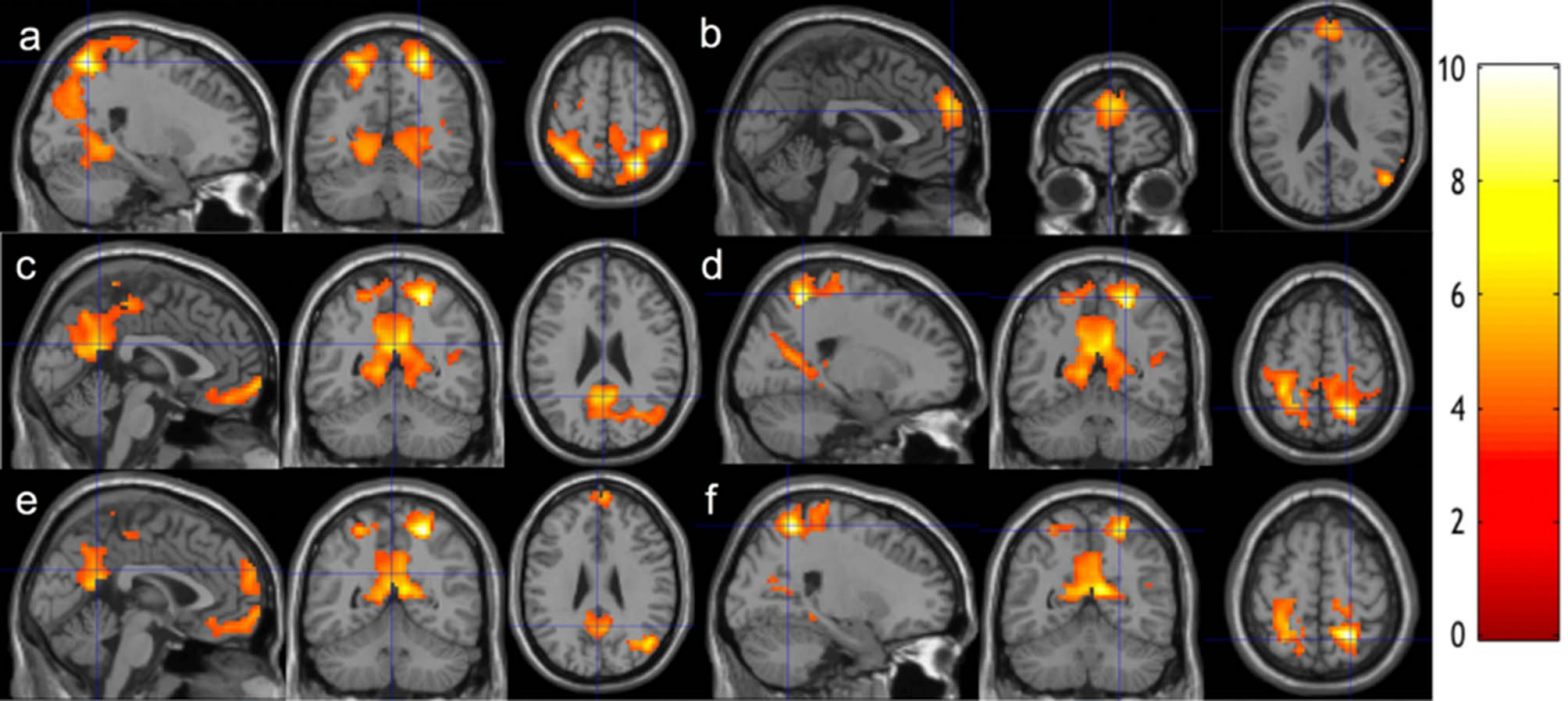
Increased functional connectivity from baseline to follow-up**.** Regions overlaid on a standard brain template in which significantly increased functional connectivity was observed with a seed ROI at the **(a)** PCC, **(b)** RMT, **(c,d)** RSPL, **(e,f)** LSPL regions after 2-month meditation training at the thresholds of a voxel-level p-value of 0.005 and corrected cluster-level p-value of 0.05. The color bar shows the range of t-values. *PCC *posterior cingulate cortex, *RMT* right medial temporal cortex, *RSPL* right superior parietal lobule, *LSPL* left superior parietal lobule.

**Figure 2:**
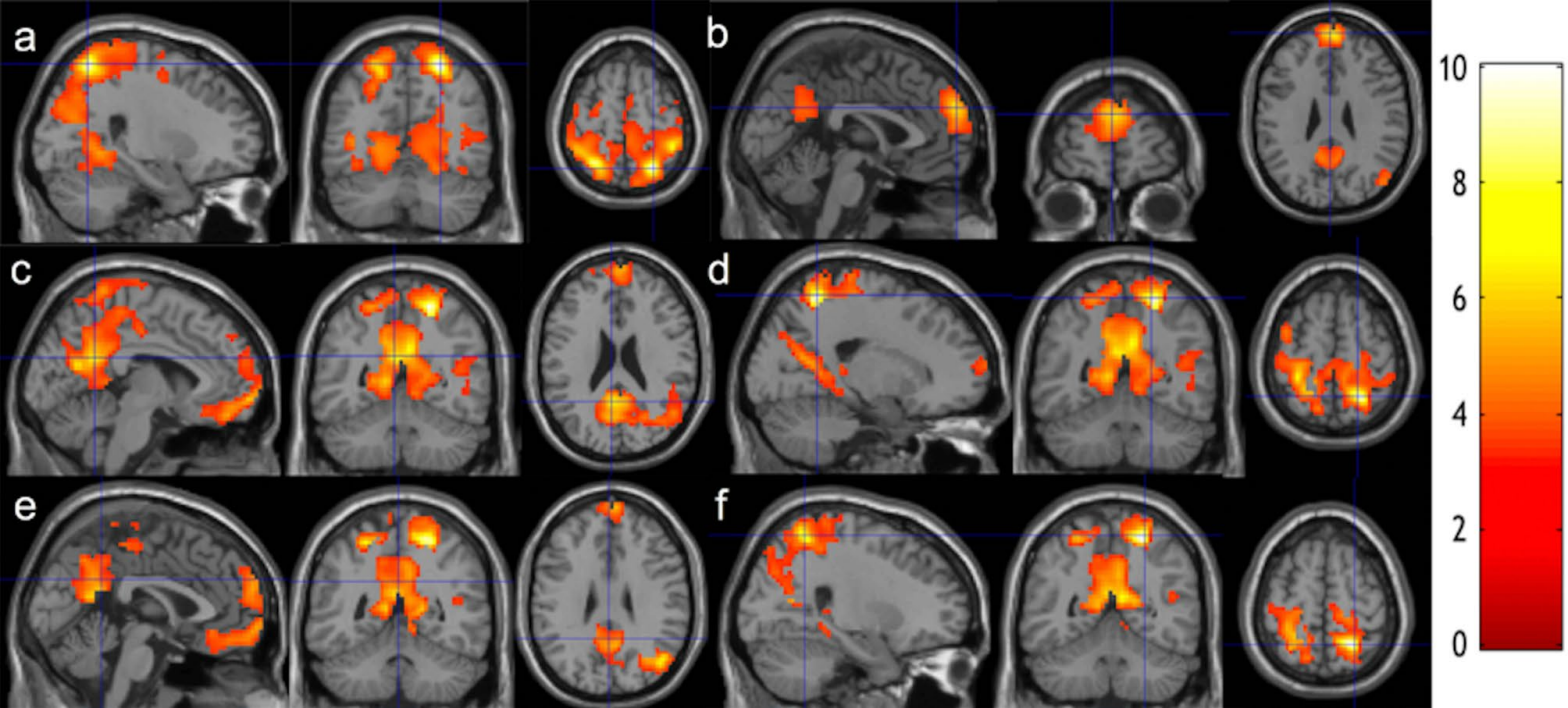
Increased functional connectivity from baseline to follow-up**.** Regions overlaid on a standard brain template in which significantly increased functional connectivity was observed with a seed ROI at the **(a)** PCC, **(b)** RMT, **(c,d)** RSPL, **(e,f)** LSPL regions after 2-month meditation training at the thresholds of a voxel-level p-value of 0.01 and corrected cluster-level p-value of 0.05. The color bar shows the range of t-values. *PCC* posterior cingulate cortex, *RMT* right medial temporal cortex, *RSPL* right superior parietal lobule, *LSPL* left superior parietal lobule.

After meditation training, we observed longitudinally increased functional connectivity between the posterior cingulate cortex (PCC) seed and the bilateral inferior parietal regions (Fig. 1a), the right middle temporal (RMT) seed and the superior medial frontal region (Fig. 1b), the right superior parietal lobule (RSPL) seed and the precuneus/PCC region (Fig. 1c), the left superior parietal lobule (LSPL) seed and the precuneus/PCC region (Fig. 1e), the RSPL seed and the bilateral inferior parietal regions (Fig. 1d), and the LSPL seed and the bilateral inferior parietal regions (Fig. 1f) at a voxel-level p-value of 0.005, using the BOLD signal time series with multi-echo independent component analysis (ME-ICA) denoising. Cluster level statistics for all the significant clusters are shown in Table 2. For purposes of illustration, this group comparison was then repeated after lowering the voxel-level threshold to p < 0.01 to seek network-level regional extents. This comparison showed significantly increased functional connectivity between the PCC seed and the DAN (Fig. 2a), the RMT seed and the DMN (Fig. 2b, although the left lateral posterior region was missed), the RSPL seed and the DMN (Fig. 2c, although the left lateral posterior region was missed), the RSPL seed and the DAN (Fig. 2d), the LSPL seed and the DMN (Fig. 2e, although the left lateral posterior region was missed), the LSPL seed and the DAN (Fig. 2f). Taken together, the 2-month meditation significantly increased the rsFC within the DAN, between the DMN and the DAN, and between the DMN and the VC. The clusters remain significant after including handedness as an additional covariate.

**Table 2:** Summary of cluster-level statistics for clusters showing significant longitudinal changes after meditation practice.

	N voxels	Z score	Peak-t MNI coordinates	Anatomical locations	% cluster	% region
Increased rsFC at PCC (Fig. 1a)	5737	4.63	24, − 62, 61	**Parietal lobe**		
				Postcentral_R	9.87	48.36
				Parietal_Sup_R	7.50	63.21
				Parietal_Sup_L	6.14	55.68
				Postcentral_L	5.44	26.18
				Precuneus_L	3.96	21.02
				Paracentral_Lobule_R	2.58	57.82
				Precuneus_R	2.20	12.60
				Parietal_Inf_L	2.07	15.88
				Paracentral_Lobule_L	1.43	19.85
				Parietal_Inf_R	1.19	16.51
				**Temporal lobe**		
				Temporal_Sup_R	5.42	32.34
				Temporal_Mid_R	2.49	10.59
				Heschl_R	1.03	77.39
				**Occipital lobe**		
				Cuneus_R	4.32	56.88
				Lingual_L	3.92	35.08
				Lingual_	3.77	30.67
				Cuneus_L	3.75	46.02
				Occipital_Mid_R	3.42	30.51
				Occipital_Sup_L	2.74	37.54
				Occipital_Mid_L	2.72	15.58
				Occipital_Sup_R	2.58	34.21
				Calcarine_R	1.78	17.90
				Calcarine_L	1.66	13.74
				Fusiform_R	1.43	10.64
				Occipital_Inf_R	1.01	19.15
				**Frontal lobe**		
				Precentral_R	4.25	23.57
				Rolandic_Oper_R	2.96	41.72
				**Insula**		
				Insula_R	2.21	23.44
	313	3.94	− 47, − 38, 28	**Temporal lobe**		
				Temporal_Sup_L	45.37	20.20
				**Insula**		
				Insula_L	2.88	1.58
				**Parietal lobe**		
				SuperMarginal_L	39.30	31.99
				Postcentral_L	3.19	0.84
				**Frontal lobe**		
				Rolandic_Oper_L	8.63	8.91
	589	3.56	− 59, 1, 34	**Parietal lobe**		
				Postcentral_L	43.12	21.32
				**Frontal lobe**		
				Precentral_L	42.78	23.34
				Frontal_Inf_Oper_L	6.79	12.59
				Frontal_Sup_L	2.89	1.54
				Rolandic_Oper_L	1.87	3.63
Increased rsFC at RMT (Fig. 1b)	332	3.84	3, 57, 28	**Frontal lobe**		
				Frontal_Sup_Medial_L	67.77	24.56
				Frontal_Sup_Medial_R	30.42	15.46
				Frontal_Sup_L	1.51	0.45
	294	3.76	53, − 70, 19	**Temporal lobe**		
				Temporal_Mid_R	74.49	16.22
				Temporal_Sup_R	8.84	2.70
				Temporal_Inf_R	1.36	0.37
				**Occipital lobe**		
				Occipital_Mid_R	7.82	3.58
				**Parietal lobe**		
				Angular_R	7.48	4.10
Increased rsFC at RSPL (Fig. 1c,d)	3004	4.70	21, − 53, 61	**Parietal lobe**		
				Precuneus_L	16.61	46.20
				Precuneus_R	11.19	33.61
				Postcentral_L	7.16	18.04
				Postcentral_R	5.93	15.21
				Parietal_Sup_R	5.43	23.96
				Paracentral_Lobule_R	4.23	49.62
				Parietal_Sup_L	2.60	12.34
				Paracentral_Lobule_L	2.10	15.25
				Angular_R	1.96	11.00
				**Frontal Lobe**		
				Precentral_L	2.53	7.04
				Precentral_R	1.73	5.02
				**Occipital lobe**		
				Calcarine_L	3.86	16.78
				Calcarine_R	3.30	17.38
				Cuneus_R	3.23	22.25
				Occipital_Mid_R	3.06	14.32
				Lingual_R	2.36	10.08
				Cuneus_L	2.20	14.13
				Lingual_L	2.06	9.67
				** Temporal lobe**		
				Temporal_Mid_R	3.40	7.56
				**Limbic system**		
				Cingulum_Mid_L	3.96	20.02
				Cingulum_Post_L	3.16	67.02
				Cingulum_Mid_R	2.50	11.12
	341	3.86	− 3, 66, − 7	**Frontal lobe**		
				Frontal_Med_Orb_L	31.38	48.61
				Rectus_L	21.11	27.60
				Frontal_Med_Orb_R	17.60	22.89
				Rectus_R	17.01	25.43
				Frontal_Sup_L	4.99	1.54
				Frontal_Sup_Orb_L	3.23	3.73
				Front_Sup_Medial_L	2.05	0.76
				Frontal_Sup_Orb_R	1.76	1.97
Increased rsFC at LSPL (Fig. 1e,f)	551	4.81	24, − 53, 61	**Parietal lobe**		
				Parietal_Sup_R	28.31	22.93
				Postcentral_R	23.96	11.28
				Paracentral_Lobule_R	15.43	33.21
				Precuneus_R	6.72	3.70
				Paracentral_Lobule_L	3.27	4.36
				Precuneus_L	2.54	1.30
				Parietal_Inf_R	2.54	3.40
				**Frontal lobe**		
				Precentral_R	12.16	6.47
				Supp_Motor_Area_R	3.45	2.62
				**Limbic system**		
				Cingulum_Mid_R	1.63	1.33
	1131	4.43	12, − 59, 16	**Occipital lobe**		
				Occipital_Mid_R	9.55	26.81
				Calcarine_L	7.25	11.86
				Calcarine_R	5.31	10.53
				Occipital_Sup_R	2.48	6.47
				Cuneus_L	2.12	5.14
				Cuneus_R	1.24	3.21
				Lingual_R	3.89	6.25
				**Limbic system**		
				Cingulum_Post_L	7.52	59.96
				Cingulum_Mid_R	3.80	6.38
				Cingulum_Mid_L	2.30	4.38
				Cingulum_Post_R	1.68	18.52
				ParaHippocampal_R	1.33	4.33
				**Temporal lobe**		
				Temporal_Mid_R	1.06	0.89
				**Parietal lobe**		
				Precuneus_L	21.22	22.22
				Preceneus_R	18.04	20.42
				Angular_R	5.92	12.49
				Parietal_Sup_R	1.06	1.76
				**Cerebelum**		
				Cerebelum_4_5_R	1.95	8.35
	582	4.00	12, 60, − 16	**Frontal lobe**		
				Frontal_Sup_Medial_L	22.85	14.52
				Frontal_Med_Orb_L	16.15	42.70
				Rectus_L	12.89	28.75
				Frontal_Med_Orb_R	11.00	24.42
				Frontal_Sup_Medial_R	11.00	9.80
				Frontal_Sup_Orb_R	8.42	16.05
				Rectus_R	7.90	20.17
				Frontal_Sup_L	6.87	3.63
				Frontal_Sup_Orb_L	2.06	4.07
	380	3.91	− 24, − 50, 58	**Parietal lobe**		
				Postcentral_L	43.68	13.93
				Parietal_Sup_L	21.32	12.81
				Precuneus_L	12.63	4.44
				Paracentral_Lobule_L	4.47	4.12
				Parietal_Inf_L	4.21	2.14
				**Frontal lobe**		
				Precentral_L	12.89	4.54
Increased rsFC at LSPL associated with practice time (Fig. 4)	259	4.15	− 6, 42, 31	**Limbic system**		
				Cingulum_Ant_L	39.77	24.03
				Cingulum_Ant_R	19.69	12.69
				**Frontal lobe**		
				Frontal_Sup_Medial_L	35.52	10.04
				Frontal_Sup_Medial_R	4.63	1.84

*MNI*: Montreal Neurological Institute.

^†^A voxel-level statistical threshold of P < 0.005 was used.

**Figure 3:**
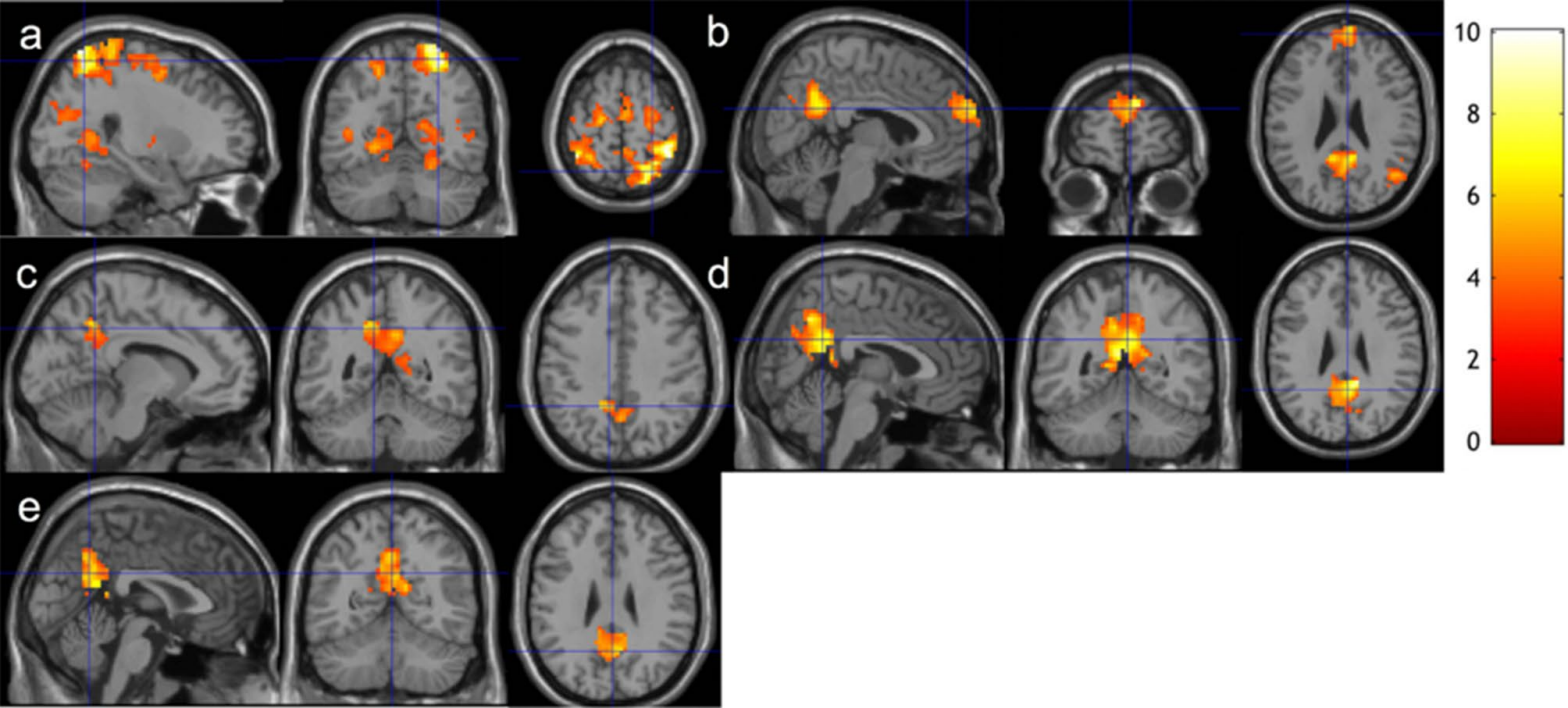
Increased functional connectivity from baseline to follow-up using the BOLD signal time series with conventional denoising**.** Regions overlaid on a standard brain template in which significantly increased functional connectivity was observed with a seed ROI at the **(a)** PCC, **(b)** RMT, **(c)** LMT, **(d)** RSPL, **(e)** LSPL regions after 2-month meditation training at the thresholds of a voxel-level p-value of 0.005 and corrected cluster-level p-value of 0.05. The color bar shows the range of t-values. *PCC* posterior cingulate cortex, *RMT* right medial temporal cortex, *LMT* right medial temporal cortex, *RSPL* right superior parietal lobule, *LSPL* left superior parietal lobule.

Using the raw BOLD time series without denoising, no longitudinal change of rsFC with any seed regions of interest (ROI) was observed after meditation practice. Using the BOLD signal time series with conventional denoising, generally less extents of the rsFC changes (Fig. 3) was observed compared to the BOLD signal time series with ME-ICA denoising. Using the BOLD signal time series with conventional denoising, we observed similar changes of rsFC with the PCC and RMT seeds (Fig. 3a,b), less extent for the changes of rsFC with the LSPL (Fig. 3c) and RSPL (Fig. 3d) seeds, and more extent for the changes of rsFC with the LMT seed (Fig. 3e). We found increased LSPL/RSPL rsFC with PCC (a medial posterior node of DMN) using the BOLD signal time series with conventional denoising, but increased LSPL/RSPL rsFC with almost the entire DMN as well as DAN using the BOLD time series with ME-ICA denoising. We also noticed increased LMT rsFC with PCC using the BOLD signal time series with conventional denoising, but with no region using the BOLD time series with ME-ICA denoising.

### Association of functional connectivity with meditation practice time

**Figure 4:**
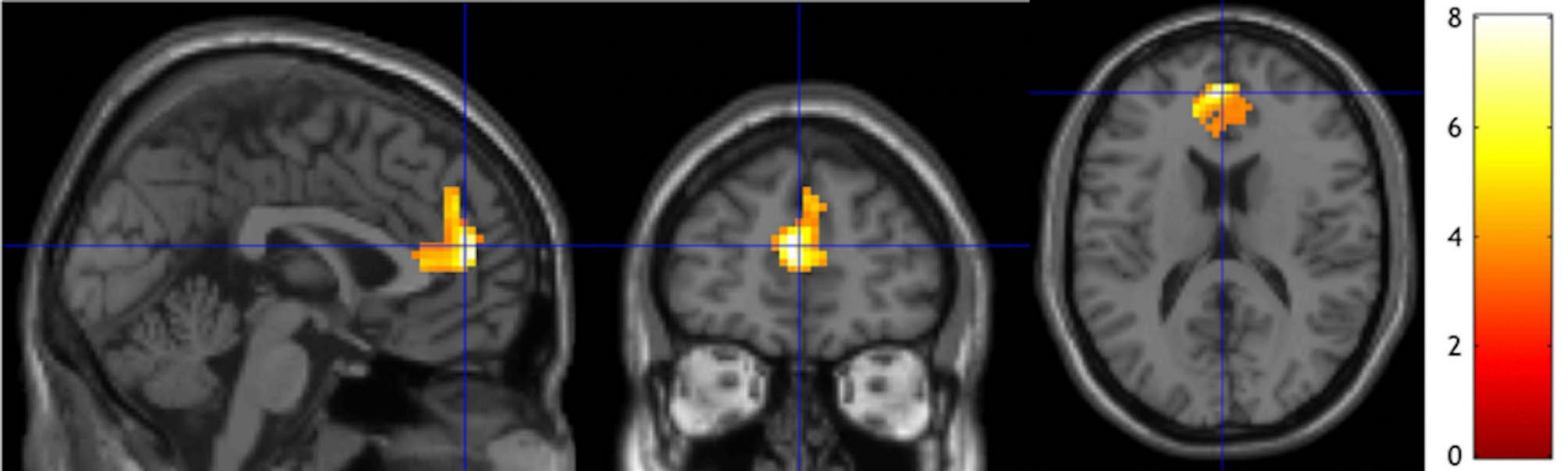
Regions of functional connectivity change associated with practice time. Regions overlaid on a standard brain template in which the meditation practice time was associated with functional connectivity with a seed ROI at the LSPL region and meditation practice time at the thresholds of a voxel-level p-value of 0.005 and corrected cluster-level p-value of 0.05. *LSPL* left superior parietal lobule.

We observed that the longitudinal changes of rsFC between the LSPL seed and medial prefrontal region (mPFC) (including anterior cingulate cortex and superior medial frontal regions) was significantly associated with individual practice time (Fig. 4), using the denoised ME BOLD time series. The association remained significant after adding handedness as an additional covariate. No significant association with meditation practice time was found for the longitudinal changes of functional connectivity with the other seeds.

### Post-hoc regional rsFC analysis

**Figure 5:**
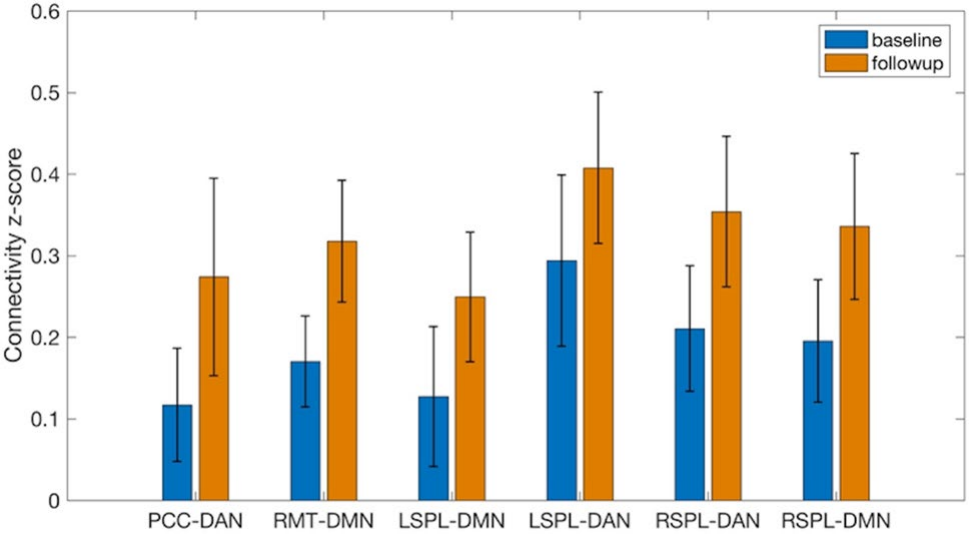
The increases of functional connectivity z-scores from baseline to follow-up. The bars stand for standard deviations. *PCC* posterior cingulate cortex, *RMT* right medial temporal region, *LSPL* left superior parietal lobe, *RSPL* right superior parietal lobe, *DAN* dorsal attention network, *DMN* default mode network.

**Figure 6:**
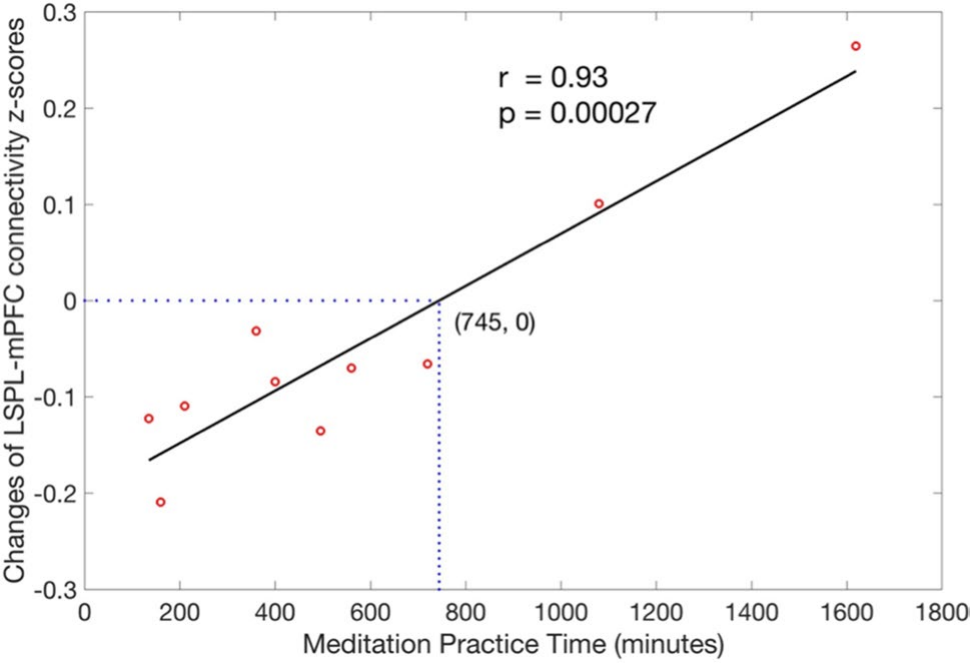
Association of longitudinal functional connectivity change with meditation practice time after adjusting for gender effects. Association, calculated from Pearson partial correlation, was observed significant (r = 0.93, p = 0.00027) and remained significant (r = 0.94, p = 0.00053) for all the subjects.

The z-scores of rsFC at baseline and follow-up are shown in Fig. 5. All the ROIs exhibited positive z-scores both at baseline and follow-up, and markedly increased rsFC from baseline to follow-up. Post-hoc regional analysis confirmed the significantly positive association between the changes in LSPL-mPFC rsFC and meditation practice time (r = 0.93, p = 0.00027) after adjusting for the gender effects and (r = 0.94, p = 0.00053) after adjusting for both the gender and handedness effects (Fig. 6). We also observed the significantly higher rsFC change in females compared to males (p = 0.00061). Interestingly, from Fig. 6, the longitudinal changes in LSPL-ACC are not always positive. For eight participants who practiced less than 745 min (zero-crossing time), reduced LSPL-mPFC rsFC was observed (shown as negative rsFC changes). Increased LSPL-mPFC rsFC was observed for two participants who practiced longer than the zero-crossing time. These regional results indicated the direction of rsFC changes may change after a period of meditation practice.

### Longitudinal changes in gray matter (GM) density and their association with practice time

After meditation training, longitudinal changes in GM density were not significant at any regions of the brain at both voxel-levels of p < 0.005 and p < 0.01. In addition, longitudinal changes in GM density were not associated with practice time at both voxel-levels of p < 0.005 and p < 0.01.

## Discussion

The results of our study showed that 2-month meditation training increased brain functional connectivity, even when participants were not in meditative state, within the DAN, between the DMN and DAN, and between the DMN and VC, as compared to the functional connectivity at baseline. The longitudinal changes of functional connectivity between the LSPL and medial prefrontal cortex were associated with the amount of meditation practice time over a 2-month period. These findings demonstrate that FAM, regardless of each individual’s chosen object of attention, increases functional connectivity within attentional networks as well as increases connectivity across distributed brain regions serving attention, self-referential, and visual processes. The findings also demonstrate that 2-month meditation training has a significant impact on the brain functional connectivity but not on the brain structure. Therefore the observed changes in functional connectivity are solely functional changes and not related to structural changes.

The more regional extents were generally observed for the changes of rsFC using the BOLD signal time series with ME-ICA denoising than with the conventional denoising. We postulate that more extents of the rsFC changes reflect a highly effective ME-ICA processing pipeline in mitigating imaging artifacts due to subject motion and physiological noises such as cardiac and respiratory pulsations^35,36^. Further analysis from finger-tip pulse oximeters (monitored during the ME-BOLD acquisitions) showed a trend of reduced heart rate (p = 0.093) after 2-month meditation training. The changes of heart rate may further underscore the importance of quality denoising in physiological noises because the changes may influence detection of BOLD signal and thereby estimates of functional connectivity differently at baseline and follow-up. The enhancement of ME-BOLD in the meditation study is consistent with the benefits of ME-BOLD demonstrated in signal-to-noise ratio^32^, specifically in high susceptibility regions in orbitofrontal and inferior temporal cortex^36^, and in brain development^35^.

The longitudinal increase in rsFC between the DMN and DAN after meditation practice extends the current literature that reports the stronger rsFC during meditation between the DMN and DAN for experienced meditators (5.6 $\pm$ 4.2 year of meditation practice)^24^. The finding is also in line with increased connectivity between certain regions of the DMN and certain regions of the attentional networks^26,27^. For instance, Brewer et al. found the increased connectivity between the PCC and dorsal anterior cingulate cortex (dACC) during meditative state in experienced meditators (10,565 $\pm$ 5148 h of meditation practice) compared with their matched controls^26^. Kilpatrick et al. reported greater correlation between the dorsal medial prefrontal cortex (dmPFC) and the auditory/salience region^27^ in 8-week meditators (8 weekly 150-min group lessons) relative to controls. Several other studies, however, found decreased connectivity or stronger anticorrelation between the DMN and attentional networks (e.g., salience network)^28,29^. We speculate that the discrepancies may be attributed to the analytical approach (e.g., certain approach regresses out the global signal fluctuations, but others do not), specific regions examined, meditation type (e.g., focused attention or open monitoring or non-dual awareness), meditation state (e.g., during resting state or meditative state), and meditation practice time (either novice meditators or experienced meditators). For example, Josipovic et al. studied experienced meditators (4000–37,000 h of practice time) and regressed out global signal fluctuations. They reported stronger anticorrelation between the DMN and extrinsic network (including dorsal attention network) during focused-attention meditation but weaker correlation during non-dual awareness meditation^28^. The reported anticorrelation may be caused by their regressing out the global signal fluctuations. The anticorrelation (change of polarity) between brain networks after regressing out the global signals has been reported in both resting-state BOLD and ASL studies^37^.

We found that rsFC between the mPFC (part of DMN, see Fig. 4) and the LSPL seed (part of DAN) decreased within less than 745-min meditation practice time regime (see Fig. 6). These results are consistent with lower connectivity between the DMN and salience attention network (or visual network) after 8-week FAM (700 $\pm$ 12.89 min) for novice elderly meditators^29^. In fact, brain neural activity with a stimulus task has been shown as an inverted U shape curve with increased meditation experience^12^. The nonlinear correlation of brain neural activity with meditation experience may partly confound the direction of the changes of functional connectivity.

The increased rsFC between the DMN and DAN and between the DMN and VC were generally observed. Please note that the LSPL seed (part of DAN) was shown with consistently increased rsFC with anterior mPFC (part of DMN, Fig. 1e) but with decreased rsFC with posterior mPFC within less than 745-min meditation practice time regime (Fig. 4). The increased rsFC suggests greater synergy coherence between self-referential, attention, and visual processing and may facilitate switching between the networks^38^. The shift to stronger functional connectivity can reflect the practice of FAM, which requires realizing that the attention has been distracted from the chosen object of meditation (such as the sensation of breathing or the sound of metronome) to self-relevant feeling, thoughts, and memory, releasing the distraction, and reorienting attention back to the intended meditation object. The process of meditation involves attentional regulation and self-monitoring. Therefore, meditation practice may require functional coupling of the DAN and the DMN to coordinate this complex process. These brain networks were shown to be more functionally coupled during the meditative state^24,26,27^. Our results suggest that the increased coupling during the meditative state may carry over to the rest state with repetitive meditation practice (i.e., a trait of a brain function instead of a state).

Previous cross-sectional studies reported that experienced meditators had elevated rsFC within the DAN compared to controls^24^ and that novice meditators exhibited increased msFC within the DAN compared to controls^27^. Using the longitudinal design, we confirmed that the strengthened rsFC within the DAN is indeed caused by the meditation experience. This finding may reflect that meditation inhibits distracting the self-referential thoughts and maintains focused attention. It has been shown that experienced meditators exhibited more activation in attention-related brain regions when they performed tasks involving conflict monitoring or distracting sounds^11,12,39^. It is plausible that these increases in rsFC are likely to underlie increased attention control stemming from repetitive meditation experience.

The practice time was significantly correlated with the rsFC change between the LSPL seed of the DAN and the mPFC of the DMN. This positive association is supported by the association between the number of years of meditation experience and rsFC between the DAN and DMN^24^ in the experienced meditators. The rsFC association with practice time in that study was only at a single time point, while the association in our study was for the change of rsFC between two time points. Further analysis was performed to evaluate the association of rsFC with practice time at a single time point (either baseline or follow-up). Only at follow-up, the rsFC between the LSPL seed and mPFC was positively associated with practice time with voxel-level p of 0.005 but not significant after multiple correction. Therefore, the rsFC association with practice time at follow-up can be attributed to the change of rsFC from baseline rather than its baseline rsFC. we have extended the literature by using the longitudinal design to investigate the functional connectivity change and drawing a direct inference for the causal relationship between practice time and functional connectivity change. The rsFC change in the mPFC is also in good agreement with the previous reports for the association of structural and functional changes in the mPFC with meditation^11,12,15,21^. Experienced meditators demonstrated heightened rsFC with mPFC within DMN compared to controls^25^. Taking it together with our results that indicate meditation practice can heighten the rsFC between the mPFC with a region in DAN, we can further confirm the role of the mPFC in integrating information gathered from internal and external environments. The stronger connectivity of mPFC with the DMN and DAN from meditation practice may effectively resolve the conflicts between the two networks and therefore achieve fast switching of brain networks. These results suggest that a 2-month meditation training can lead to changes in brain functional connectivity, which may reflect cortical remodeling between brain regions.

The study is not without limitations. First, we included a relatively small sample size and different variations of FAM practice. However, the sample size was shown with sufficient power in detecting the longitudinal rsFC changes, and we were able to observe significant changes of rsFC after a 2-month meditation training, further supporting the larger effect size from the applied multi-echo fMRI method. Second, we did not include a control group. However, the longitudinal change of rsFC was observed correlating with meditation practice time. Hence, it is unlikely that the results were due to expectancy bias but rather reflected the efforts participants put into their meditation practice. Third, a short follow-up period and only one follow-up time were used in the study. As such, the results may not be generalized to long-term meditation. A study with a larger sample size, matched control group and longer follow-up is warranted to confirm the association of the changes in functional connectivity with meditation.

## Conclusion

We demonstrated increased functional connectivity within the attention network, between the default mode network and attention network, and between the default mode network and visual cortex after novice meditators practiced 2-month meditation. The findings indicate the potential effects of meditation on enhancing the brain capability of fast switching between mind wandering and focused attention and maintaining attention once in attentive state.

## Methods

### Sample size justification

After weeks of meditation training for 14 PTSD patients, mindfulness-based meditation training significantly increased the DMN rsFC (the PCC seed) with a p value of at most 0.005^31^. This p value is equivalent to a t value of 3.01 and an effect size of 0.81. Another meditation training study demonstrated the increased CEN-DAN rsFC (dorsal lateral prefrontal cortex seed) after 3-day meditation experience with a t value of at least 3.74^30^. This t value is equivalent to an effect size of 0.88 for their sample size of 18 subjects. Our adopted ME BOLD fMRI method increased the effect size at least 24% for the DMN connectivity (using the PCC seed) with the medium-to-high motion subjects^32^. Compared to conventional BOLD, ME BOLD reduced the standard deviation of PCC rsFC (estimated from their Fig. 2: 1.5 times smaller for medium motion subjects and 2 times smaller for high motion subjects) with the one sampled t test and thereby reduced standard deviation of 1.75/$\sqrt{2}$ = 1.24 with the paired t-test. For detecting a meditation effect size of 1.00 (= 0.81 $\times$ 1.24, where 0.81 is the lower effect size using conventional BOLD in the literature ) using ME BOLD between baseline and follow-up, our study would require a sample size of 9 to achieve a power of 80% and a level of significance of 5%. In this study, we had an actual sample size of 10, providing sufficient power to detect the mediation effect.

### Study population

Eleven participants (19.09 ± 0.54 years old, age range: 19 to 20 years old college students, 5 females) were recruited from a Binghamton University meditation course, which is called “Meditation—Calm, Focus, and Reason”. Two of the participants reported to have had a brief meditation period in their prior yoga classes in which no specific guidance for meditation was given. The rest of the participants had never had meditation experience before. As such, all participants were novice meditators who had an interest in practicing meditation.

The meditation course reviewed several types of meditation across multiple culture regions, explored the methods of general meditative techniques, and investigated the impact of meditation upon physical, mental, and emotional fitness via literature reviews. The instructor also provided guided meditation for 15 min once or twice per week during class in order to expose students to a range of meditation styles. Participants were allowed to choose their own object of attention during the meditation practice, for example, their breath, a point on the wall, a phrase, or anything else as they saw fit. Participants were instructed during class to sit comfortably, relax shoulders, close/open eyes, concentrate on a selected point focus (e.g., breath), and repeatedly bring the attention back to the selected focus if they noticed their attention had drifted. Homework exercise was assigned to practice FAM for a minimum of 10 min per session and at least 5 sessions per week and each session on a different day, and to write a weekly journal by describing their practice experiences.

All eleven participants attended the baseline MRI scans, which occurred prior to any homework assignments but after 4 sessions of instructor-guided in-class practice. Ten subjects completed follow-up scans after 2 months, with one female subject unable to attend. During the practice period, participants recorded their practice time for each session in a separate log. To ensure accuracy, the logs were released to the principal investigator after the course instructor released their final grades. The total practice time was calculated as the sum of all meditation practice time (during class and during homework practice, in minutes) between the baseline and follow-up scans.

### MRI acquisition

All participants (11 participants at baseline and 10 participants at the 2-month follow-up) were scanned on the same 3 T GE MR750 scanner (General Electric, Milwaukee, United States) at Cornell University MRI Facility using a 32-channel receive-only phased-array head coil. The study was approved by the Institutional Review Board of Cornell University, and all participants gave written informed consent. All methods described in this manuscript were carried out in accordance with the approved guidelines. The scan began with a three-plane localizer to define the anatomy of interest. Sagittal T1-weighted magnetization prepared rapid gradient echo (MPRAGE) images covering the whole brain were acquired in 5 min 30 s with parameters: 176 slices with matrix size: $256 \times$ 256; slice thickness: 1.0 mm, echo time (TE): 3.42 ms, repetition time (TR): 7 ms, inversion time (TI): 425 ms, flip angle: 7^0^, field of view (FOV): 25 cm, receiver bandwidth (rBW): 25 kHz. Next, the resting-state BOLD fMRI was performed. The acquisition was implemented with ME echo planar imaging (EPI) readout: FOV: 24 cm, matrix size: 64 $\times$ 64, slice thickness: 3.75 mm, TR: 3 s, TEs: 13.6, 30, 47 ms. Each TR resulted in the acquisition of 3 volumes, one for each TE. Two hundred TRs (600 BOLD images) were collected in 10 min. Cardiac cycle and respiratory cycle were measured by pulse oximeter and respiratory bellows during the same time of BOLD fMRI acquisition.

### Image processing

For each subject, ME BOLD signal time series were transformed to the standard brain space using the T1-weighted images as an intermediate. Specifically, BOLD time series and T1-weighted images were registered using 12-parameter affine registration^40^; T1-weighted images were normalized to the MNI anatomical template using nonlinear registration using FSL FNIRT^41^. The ME BOLD data was processed following the ME-ICA pipeline, detailed elsewhere^32^. ME-ICA started with a multi-echo principal component analysis (ME-PCA) on ME BOLD data to remove thermal noise, proceeded with FastICA to decompose the dimensionally reduced PCA results into independent components (ICs), classified the ICs into BOLD components and non-BOLD artifacts according to the assessments of component-level TE-dependence, and finally combined the BOLD components to form optimally combined time series. The images from the first four TRs were discarded for increased stability. Motion artifacts were regressed out from the remaining optimally combined BOLD time series with linear regression models using six rigid-body parameters of translation and rotation (by referencing to the first BOLD volume). The BOLD signal time series with ME-ICA denoising were obtained for further analysis.

To test the effectiveness of the ME-ICA denoising pipeline, for comparison purpose, we also calculated the raw BOLD signal time series without denoising and the BOLD signal time series with conventional denoising. The raw BOLD signal time series was generated by averaging ME BOLD images with TE-dependent weights. The BOLD signal time series with conventional denoising preprocessed the raw BOLD time series by regressing out the white matter and CSF signal fluctuations and motion effects (6-parameter rigid body motion) and applying a band-pass filter [0.01, 0.08] Hz.

The three BOLD signal time series was used separately to calculate rsFC maps. Based on our hypotheses, five ROIs were chosen from the DMN, DAN, and VC. The locations of the seeds were selected from the previous work^42^: the PCC (MNI coordinate [1, − 55, 17] mm) for the DMN, the LMT (MNI coordinate [− 45, − 69, − 2] mm) and RMT (MNI coordinate [50, − 69, − 3] mm) for the VC, the LSPL (MNI coordinate [− 27, − 52, 57] mm) and RSPL (MNI coordinate [24, − 56, 55] mm) for the DAN. All seed ROIs were defined as a sphere with a volume of ~ 2 cm^3^ centered at each seed voxel. These regions were then used as seed regions to create functional correlation maps in the datasets. The rsFC map for each volunteer and each seed ROI were calculated using Pearson correlation coefficients between time series from the seed ROI and those from the voxels throughout the entire brain. The time series from each seed ROI was calculated as the mean time series across all the voxels within the seed ROI.

For each of BOLD signal time series, the five functional connectivity maps from each participant were then transformed into z-score maps by using a Fisher z-transformation in order to achieve a normal distribution of z-scores at each voxel over the population for group-level statistics. The z-score maps were further smoothed with a 6 × 6 × 6 mm Gaussian kernel.

To assess whether the changes of rsFC reflect the underlying structural changes, we performed a voxel-based morphometry (VBM) analysis to evaluate the brain structural changes from meditation. The Diffeomorphic Anatomical Registration Through Exponentiated Lie Algebra (DARTEL) method has been used to achieve more accurate inter-subject registration of brain structural images. We adopted the DARTEL-based VBM analysis using SPM12. First, T1-weighted images were segmented into GM and WM probability maps and transformed into the MNI space, which were initial import in DARTEL. Second, the procedure of Run DARTEL (create Templates) was performed to estimate the deformations that best align the GM and WM images together. Specifically, an initial template was created from the imported GM and WM images. The first iteration of registration warped each image to the initial template using the DARTEL approach, following by generating a new template by the average of the registered images. The registered images were iteratively updated using the template created in the previous run. The step resulted in six templates and a flow field map that parameterized the deformation for every image. Third, the procedure of Normalized to MNI Space and smoothing was performed. The flow field map (i.e., deformations estimated in the previous step) was applied to the GM images to create spatially normalized GM images in the MNI space. A Gaussian smoothing kernel of 6 mm was applied to the normalized GM images.

### Statistical analysis

In order to assess the changes of rsFC between baseline and follow-up at the group level, the z-score maps were compared using SPM12 via a paired t-test with gender as a covariate on a voxel-by-voxel basis. Age of the participants was not considered as a covariate because the maximum difference among their ages is only 1 year. After the initial models, handedness was included as an additional covariate to evaluate its effect on the changes of rsFC. The statistical maps were thresholded at a voxel-level p-value of 0.005. A cluster-level p-value of 0.05 was used to correct for multiple comparisons. The automated anatomical labeling (AAL) atlas binary mask was used to include only the significant clusters within the gray matter area. A more liberal voxel-level threshold of p < 0.01 was used to view the trends for extended significant regions, although a corrected cluster-level threshold of p < 0.05 was still used for comparisons. Similar to the analyses in the rsFC images, the GM density images were compared between baseline and follow-up via a paired t-test with gender as a covariate; the association of the longitudinal changes in GM density with practice time was modeled via a multiple linear regression with gender and meditation practice time as covariates.

To investigate the association of the longitudinal rsFC changes with total meditation practice time, the difference of the z-score maps between baseline and follow-up was modeled using SPM12 on a voxel basis via multiple linear regression with gender and meditation practice time as covariates. The voxel-level p-value and corrected cluster-level p-value thresholds were set in the same way as those in the aforementioned group comparison of rsFC maps between baseline and follow-up. Because the BOLD signal time series with ME-ICA denoising were demonstrated more effective in detecting the longitudinal rsFC changes, the association analyses and later post-hoc regional analyses were investigated using only the BOLD signal time series with ME-ICA denoising.

Post-hoc regional analyses were performed to visualize the longitudinal rsFC changes. The significant clusters obtained from the voxel-based longitudinal rsFC changes were separated into different ROIs. According to locations of brain resting-state networks, several regions were combined into ROIs for the network analyses, and the ROIs were named with seed-network conventions. For instance, PCC-DAN stands for the within-the-DAN clusters, which exhibited significant changes with PCC rsFC. Post-hoc regional analyses were also performed to visualize the relationship between the longitudinal rsFC changes and meditation practice time. The longitudinal rsFC changes in the ROIs (that were derived from the voxel-based analysis) were analyzed using multiple linear regression analysis with gender and meditation practice time as covariates. Pearson partial correlation was calculated between the longitudinal rsFC changes and meditation practice time while adjusting the effect of gender.

## Data Availability

Raw data were generated from MRI scanner. Reconstruction software is vendor’s proprietary product. Sharing of derived data will be supported by direct request. After publishing our main findings, requests for data will be evaluated on a case-by-case basis. Before sharing data we will make sure that all data are free of identifiers that could directly or indirectly link information to an individual and that all sharing is compliant with institutional and IRB policies.

## References

[1] Mooneyham BW, Mrazek MD, Mrazek AJ, Schooler JW (2016). Signal or noise: Brain network interactions underlying the experience and training of mindfulness. Ann. N. Y. Acad. Sci..

[2] Mason MF (2007). Wandering minds: The default network and stimulus-independent thought. Science.

[3] Raichle ME (2001). A default mode of brain function. Proc. Natl. Acad. Sci. U S A.

[4] Christoff K, Gordon AM, Smallwood J, Smith R, Schooler JW (2009). Experience sampling during fMRI reveals default network and executive system contributions to mind wandering. Proc. Natl. Acad. Sci. U S A.

[5] Simpson, J. R., Jr., Drevets, W. C., Snyder, A. Z., Gusnard, D. A. & Raichle, M. E. Emotion-induced changes in human medial prefrontal cortex: II. During anticipatory anxiety. *Proc. Natl. Acad. Sci. U S A* **98**, 688–693.:10.1073/pnas.98.2.688 (2001). 10.1073/pnas.98.2.688 PMC14649 11209066

[6] Andrews-Hanna JR, Reidler JS, Sepulcre J, Poulin R, Buckner RL (2010). Functional-anatomic fractionation of the brain's default network. Neuron.

[7] Ptak R, Schnider A (2010). The dorsal attention network mediates orienting toward behaviorally relevant stimuli in spatial neglect. J. Neurosci..

[8] Corbetta M, Patel G, Shulman GL (2008). The reorienting system of the human brain: From environment to theory of mind. Neuron.

[9] Vossel S, Geng JJ, Fink GR (2014). Dorsal and ventral attention systems: Distinct neural circuits but collaborative roles. Neuroscientist.

[10] Parks EL, Madden DJ (2013). Brain connectivity and visual attention. Brain Connect.

[11] Holzel BK (2007). Differential engagement of anterior cingulate and adjacent medial frontal cortex in adept meditators and non-meditators. Neurosci. Lett..

[12] Brefczynski-Lewis JA, Lutz A, Schaefer HS, Levinson DB, Davidson RJ (2007). Neural correlates of attentional expertise in long-term meditation practitioners. Proc. Natl. Acad. Sci. U S A.

[13] Farb NA (2007). Attending to the present: Mindfulness meditation reveals distinct neural modes of self-reference. Soc. Cogn. Affect. Neurosci..

[14] Goldin PR, Gross JJ (2010). Effects of mindfulness-based stress reduction (MBSR) on emotion regulation in social anxiety disorder. Emotion.

[15] Holzel BK (2008). Investigation of mindfulness meditation practitioners with voxel-based morphometry. Soc. Cogn. Affect. Neurosci..

[16] Vestergaard-Poulsen P (2009). Long-term meditation is associated with increased gray matter density in the brain stem. NeuroReport.

[17] Hernandez SE, Suero J, Barros A, Gonzalez-Mora JL, Rubia K (2016). Increased grey matter associated with long-term Sahaja yoga meditation: A voxel-based morphometry study. PLoS ONE.

[18] Grant JA (2013). Cortical thickness, mental absorption and meditative practice: Possible implications for disorders of attention. Biol. Psychol..

[19] Grant JA, Courtemanche J, Duerden EG, Duncan GH, Rainville P (2010). Cortical thickness and pain sensitivity in Zen meditators. Emotion.

[20] Yang CC (2019). Alterations in brain structure and amplitude of low-frequency after 8 weeks of mindfulness meditation training in meditation-naive subjects. Sci. Rep..

[21] Lazar SW (2005). Meditation experience is associated with increased cortical thickness. NeuroReport.

[22] Holzel BK (2010). Stress reduction correlates with structural changes in the amygdala. Soc. Cogn. Affect. Neurosci..

[23] Holzel BK (2011). Mindfulness practice leads to increases in regional brain gray matter density. Psychiatry Res..

[24] Froeliger B (2012). Meditation-state functional connectivity (msFC): Strengthening of the dorsal attention network and beyond. Evid. Based Complement. Alternat. Med..

[25] Jang JH (2011). Increased default mode network connectivity associated with meditation. Neurosci. Lett..

[26] Brewer JA (2011). Meditation experience is associated with differences in default mode network activity and connectivity. Proc. Natl. Acad. Sci. U S A.

[27] Kilpatrick LA (2011). Impact of mindfulness-based stress reduction training on intrinsic brain connectivity. Neuroimage.

[28] Josipovic Z, Dinstein I, Weber J, Heeger DJ (2011). Influence of meditation on anti-correlated networks in the brain. Front. Hum. Neurosci..

[29] Cotier FA, Zhang R, Lee TMC (2017). A longitudinal study of the effect of short-term meditation training on functional network organization of the aging brain. Sci. Rep..

[30] Taren AA (2017). Mindfulness meditation training and executive control network resting state functional connectivity: A randomized controlled trial. Psychosom. Med..

[31] King AP (2016). Altered default mode network (Dmn) resting state functional connectivity following a mindfulness-based exposure therapy for posttraumatic stress disorder (Ptsd) in combat veterans of Afghanistan and Iraq. Depress. Anxiety.

[32] Kundu P (2013). Integrated strategy for improving functional connectivity mapping using multiecho fMRI. Proc. Natl. Acad. Sci. U S A.

[33] Poser BA, Versluis MJ, Hoogduin JM, Norris DG (2006). BOLD contrast sensitivity enhancement and artifact reduction with multiecho EPI: Parallel-acquired inhomogeneity-desensitized fMRI. Magn. Reson. Med..

[34] Tang YY (2009). Central and autonomic nervous system interaction is altered by short-term meditation. Proc. Natl. Acad. Sci. U S A.

[35] Kundu P (2015). Robust resting state fMRI processing for studies on typical brain development based on multi-echo EPI acquisition. Brain Imaging Behav..

[36] Markello RD, Spreng RN, Luh WM, Anderson AK, De Rosa E (2018). Segregation of the human basal forebrain using resting state functional MRI. Neuroimage.

[37] Zhao L, Alsop DC, Detre JA, Dai W (2019). Global fluctuations of cerebral blood flow indicate a global brain network independent of systemic factors. J. Cereb. Blood Flow Metab..

[38] Seeley WW (2007). Dissociable intrinsic connectivity networks for salience processing and executive control. J. Neurosci..

[39] van Veen V, Cohen JD, Botvinick MM, Stenger VA, Carter CS (2001). Anterior cingulate cortex, conflict monitoring, and levels of processing. Neuroimage.

[40] Saad ZS (2009). A new method for improving functional-to-structural MRI alignment using local Pearson correlation. Neuroimage.

[41] Klein A (2009). Evaluation of 14 nonlinear deformation algorithms applied to human brain MRI registration. Neuroimage.

[42] Vincent JL, Kahn I, Snyder AZ, Raichle ME, Buckner RL (2008). Evidence for a frontoparietal control system revealed by intrinsic functional connectivity. J. Neurophysiol..

